# A Prospective Evaluation of Tocilizumab in Patients With Rheumatoid Arthritis Refractory to Conventional Disease-Modifying Anti-Rheumatic Medications (DMARDs)

**DOI:** 10.7759/cureus.92922

**Published:** 2025-09-22

**Authors:** Jazba Yousaf, Maria Khurshid, Sajid Naseem, Rehan Wani, Murad Ali, Ammarah Amjad, Abdullah Elrefae, Khawaja Faizan Ejaz, Miqdad Qandeel, Muhammad Iftikhar Khattak, Shahid Khan

**Affiliations:** 1 Department of Geriatric Medicine, Russells Hall Hospital, Dudley, GBR; 2 Department of Pharmacology, Azad Jammu and Kashmir Medical College, Muzaffarabad, PAK; 3 Department of Rheumatology, Fazaia Medical College, Islamabad, PAK; 4 Department of Trauma and Orthopaedics, Abbas Institute of Medical Sciences, Muzaffarabad, PAK; 5 Department of Trauma Orthopaedics, Ayub Teaching Hospital, Abbottabad, PAK; 6 Department of Pharmacology, HBS Medical and Dental College, Rawalpindi, PAK; 7 Department of Trauma and Orthopaedics, Al Bashir Hospital, Amman, JOR; 8 Department of Medicine, Russells Hall Hospital, Dudley, GBR; 9 Department of Trauma and Orthopaedics, Central Middlesex Hospital, London, GBR; 10 Department of Research and Development, Celestial and Dimanche, Muzaffarabad, PAK; 11 Department of Family Medicine, Holy Family Hospital, Rawalpindi, PAK

**Keywords:** biologic dmards, das28, il-6 inhibitors, refractory ra, rheumatoid arthritis, tocilizumab

## Abstract

Background: Rheumatoid arthritis (RA) refractory to conventional synthetic disease-modifying anti-rheumatic medications (DMARDs) remains a therapeutic challenge, necessitating the use of biologic agents such as tocilizumab.

Objective: To prospectively evaluate the efficacy of tocilizumab in reducing disease activity, measured by DAS28 (ESR), and to assess its safety in terms of adverse events over a six-month follow-up in a real-world South Asian cohort.

Methodology: This prospective observational study was conducted at the Department of Rheumatology, Abbas Institute of Medical Sciences, Muzaffarabad, over a two-year period (June 2022-May 2024). A total of 192 patients meeting the 2010 ACR/EULAR RA criteria, with inadequate response to at least two csDMARDs, including methotrexate and a baseline DAS28 (ESR) ≥5.1, were enrolled. Tocilizumab (4-8 mg/kg) was administered intravenously every four weeks for six months. DAS28 scores were recorded at baseline, 12 weeks, and 24 weeks. Safety was assessed through serial laboratory monitoring and systematic documentation of adverse events.

Results: Of the 192 patients (77.08% female; mean age 44 ± 10.6 years), the mean DAS28 score decreased from 5.91 ± 0.42 at baseline to 3.76 ± 0.59 at 12 weeks and 2.43 ± 0.71 at 24 weeks (p < 0.001). Remission (DAS28 ≤ 2.6) was achieved in 112 patients (58.33%), while 58 (30.21%) showed partial response and 22 (11.46%) had no response. Adverse events were reported in 52 patients (27.08%), most commonly infections, lipid abnormalities, and gastritis.

Conclusion: Tocilizumab is a highly effective and generally well-tolerated treatment option for RA patients unresponsive to csDMARDs, though longer follow-up and controlled comparisons are needed to confirm long-term safety and causality.

## Introduction

Rheumatoid arthritis (RA) is a long-term inflammatory disease that affects the whole body and causes ongoing inflammation of the synovial fluid, damage to the joints over time, and loss of function [1]. About 0.5% to 1% of people throughout the world have it, and it has a bigger effect on women and people in their most productive years [2]. The development of RA is caused by a complicated mix of genetic, environmental, and immunological variables that cause the immune system to attack self-antigens, which leads to joint damage and other symptoms throughout the body [3].

The main way to treat RA is with disease-modifying anti-rheumatic medications (DMARDs). The first-line medicines are conventional synthetic DMARDs (csDMARDs) such as methotrexate, sulfasalazine, and hydroxychloroquine [4]. The goal of these medications is to minimize inflammation, protect joints from harm, and keep them working [5]. However, a large number of patients do not attain long-term disease management with csDMARDs because they do not respond well or have side effects that are too bad to handle [6]. These people have refractory RA, which means they have a lot of pain and problems with their health care, and their quality of life is worse [7].

Biologic DMARDs (bDMARDs) that target certain immune pathways have become a game-changing alternative for people with RA that doesn't respond to other treatments in the last few years [8]. Tocilizumab, a humanized monoclonal antibody that targets the interleukin-6 (IL-6) receptor, has been shown to be very effective [9]. IL-6 is a key cytokine in the inflammatory cascade of RA. It causes synovitis, joint destruction, and systemic symptoms [10]. Tocilizumab is a targeted way to change immunological dysregulation in RA by blocking IL-6 signaling [11]. In both monotherapy and combination settings, clinical studies have indicated that tocilizumab greatly improves disease activity ratings, physical function, and radiographic results [12].

Even though randomized controlled studies have shown that tocilizumab works and is safe, there isn't much real-world data on how well it works and how well it is tolerated in people who don't respond to csDMARDs, especially in a variety of clinical contexts. It is very important to test tocilizumab in these groups so that personalized treatment plans may be made and long-term results can be improved. The objective of this study was to prospectively evaluate the efficacy of tocilizumab in reducing disease activity, measured by DAS28, and to assess its safety in terms of adverse events over a six-month follow-up period in a real-world South Asian cohort of patients with rheumatoid arthritis refractory to conventional synthetic DMARDs.

## Materials and methods

Study design and setting

This prospective observational study was conducted at the Department of Rheumatology, Abbas Institute of Medical Sciences (AIMS), Muzaffarabad, over a two-year period from June 2022 to May 2024. The study was designed to capture real-world clinical outcomes of tocilizumab in rheumatoid arthritis (RA) patients refractory to conventional synthetic DMARDs (csDMARDs), with standardized eligibility criteria, systematic follow-up, and consistent monitoring protocols to enhance reliability and reduce variability.

Inclusion and exclusion criteria

Eligible patients were 18 years or older with a confirmed diagnosis of RA based on the 2010 ACR/EULAR classification criteria. Participants were required to have a disease duration of at least one year and a Disease Activity Score in 28 joints (DAS28) ≥5.1 despite treatment with at least two csDMARDs, including methotrexate. Patients were excluded if they had leukocyte counts <4000/cmm, platelet counts <150,000/cmm, or serious uncontrolled comorbidities such as diabetes, active infections, or hepatic/renal impairment. Additional exclusion criteria included a history of malignancy, tuberculosis, autoimmune diseases other than RA, prior exposure to tocilizumab or other IL-6 inhibitors, use of high-dose corticosteroids (>0.5 mg/kg/day), and pregnancy or lactation. These strict criteria were applied to minimize confounding and ensure patient safety.

Sample size and sampling technique

A total of 208 patients were initially enrolled, of whom 16 (7.7%) were lost to follow-up due to withdrawal of consent, missed visits, or non-compliance, leaving 192 patients for final analysis. Patients were recruited using convenience sampling from consecutive eligible outpatients at AIMS.

An a priori power calculation was performed using G*Power version 3.1. Assuming a medium effect size (Cohen’s d = 0.5), α = 0.05, power (1-β) = 0.80, and a paired t-test to compare DAS28 scores pre- and post-treatment, the minimum required sample size was 34 patients. With 192 patients completing the study, statistical power exceeded 95%, ensuring robust detection of clinically meaningful changes in disease activity.

Although convenience sampling may introduce selection bias, the consecutive recruitment strategy reduced this risk, and the achieved sample size was substantially larger than the calculated requirement. Furthermore, it is consistent with sample sizes reported in other real-world studies on biologic therapies in refractory RA populations, such as Kamal et al. [13] and Farah et al. [14].

Dosage and concomitant medications

All patients received intravenous tocilizumab (4-8 mg/kg) every four weeks. To minimize infusion-related reactions, each infusion was preceded by an antihistamine and 250 mg of hydrocortisone. Concomitant therapy with methotrexate (10-15 mg/week) and low-dose prednisolone (<15 mg/day) was continued throughout the study. Non-steroidal anti-inflammatory drugs (NSAIDs) were permitted for symptomatic relief, provided dosing remained stable during the study.

Data collection

Baseline demographic and clinical characteristics, including age, sex, disease duration, comorbidities, and prior csDMARD use, were recorded at enrollment. Laboratory assessments included complete blood count, liver and kidney function tests, and lipid profile at baseline and repeated every four weeks.

Disease activity was measured using DAS28 at baseline, week 12 (after the third infusion), and week 24 (after the sixth infusion). A DAS28 score >4 indicated non-response, 2.7-4 indicated partial response, and ≤2.6 indicated remission.

Safety assessments included monitoring for adverse events (AEs) at each follow-up visit. Early infusion-related reactions (within 24 hours), such as hypersensitivity, were documented, along with late AEs, including infections requiring intravenous antibiotics, headaches, mucosal ulcers, and gastrointestinal intolerance. This structured AE monitoring enhanced reproducibility and minimized reporting bias.

Statistical analysis

Data analysis was conducted using IBM Corp. Released 2017. IBM SPSS Statistics for Windows, Version 26.0. Armonk, NY: IBM Corp. Continuous variables were expressed as mean ± standard deviation, and categorical variables as frequencies and percentages. The paired t-test was used to evaluate changes in DAS28 scores over time. A p-value <0.05 was considered statistically significant. The sample size achieved ensured high power, supporting the reliability of findings.

Ethical approval

The study received ethical clearance from the Institutional Review Committee of AIMS, Muzaffarabad (Approval No: 1845/AIMS/2022; dated 06-September-2022). Written informed consent was obtained from all participants prior to enrollment. All procedures adhered to the principles of the Declaration of Helsinki.

## Results

Among the 192 patients enrolled, 148 were female (77.08%) and 44 were male (22.92%), as shown in Table 1. The most common age group was 31-45 years with 76 patients (39.58%), followed by 46-60 years with 58 patients (30.21%), 18-30 years with 32 patients (16.67%), and >60 years with 26 patients (13.54%). Regarding disease duration, 79 patients (41.15%) had RA for 4-6 years, 61 (31.77%) for 1-3 years, and 52 (27.08%) for more than six years. Comorbid conditions included hypertension in 47 patients (24.48%), diabetes mellitus in 31 (16.15%), and dyslipidemia in 25 (13.02%), while 89 patients (46.35%) had no comorbidities. All patients (100%) had used methotrexate, 128 (66.67%) used sulfasalazine, 117 (60.94%) used hydroxychloroquine, and 49 (25.52%) used leflunomide, with 143 patients (74.48%) having used a combination of two or more csDMARDs.

**Table 1:** Baseline Demographic and Clinical Characteristics of Study Participants (n = 192). Data are represented as n (%).

Category	Characteristic	Patients n (%)
Age Group (years)	18–30	32 (16.67)
	31–45	76 (39.58)
	46–60	58 (30.21)
	>60	26 (13.54)
Sex	Female	148 (77.08)
	Male	44 (22.92)
Disease Duration	1–3 years	61 (31.77)
	4–6 years	79 (41.15)
	>6 years	52 (27.08)
Comorbidities	Hypertension	47 (24.48)
	Diabetes Mellitus	31 (16.15)
	Dyslipidemia	25 (13.02)
	None	89 (46.35)
Prior csDMARDs Used	Methotrexate	192 (100.0)
	Sulfasalazine	128 (66.67)
	Hydroxychloroquine	117 (60.94)
	Leflunomide	49 (25.52)
	Combination (≥2 csDMARDs)	143 (74.48)

The mean DAS28 score at baseline was 5.91 ± 0.42. After 12 weeks (third infusion), it reduced to 3.76 ± 0.59, and by 24 weeks (sixth infusion), it further declined to 2.43 ± 0.71 (Table 2). These reductions were statistically significant (p < 0.001 at both intervals), indicating marked improvement in disease activity among all 192 patients (100%).

**Table 2:** Changes in DAS28 Score Over the Study Period (n = 192). Values presented as Mean ± SD. Paired t-test was applied; p < 0.05 was considered significant.

Time Point	Mean DAS28 ± SD	p-value (vs Baseline)
Baseline	5.91 ± 0.42	–
Week 12 (3rd infusion)	3.76 ± 0.59	<0.001
Week 24 (6th infusion)	2.43 ± 0.71	<0.001

**Figure 1:**
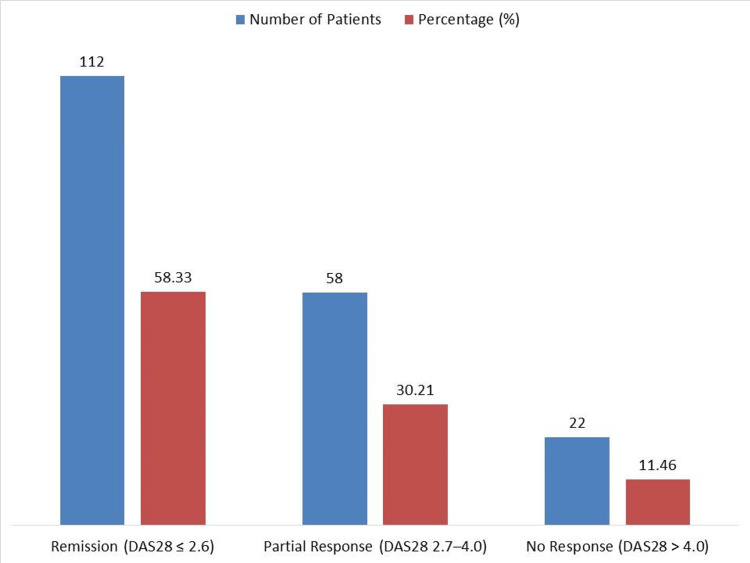
Clinical Response Categories at Six Months (n = 192). Values represented as n (%); Remission (DAS28 ≤ 2.6): 112 (58.33); Partial Response (DAS28 2.7–4.0): 58 (30.21); No Response (DAS28 > 4.0): 22 (11.46). The image is created by the author.

At six months, 112 patients (58.33%) achieved remission (DAS28 ≤ 2.6), 58 (30.21%) showed partial response (DAS28 2.7-4.0), and 22 patients (11.46%) had no response (DAS28 > 4.0), demonstrating that 170 out of 192 patients (88.54%) showed clinical benefit from tocilizumab (Figure 1).

Out of 192 patients, 52 (27.08%) experienced at least one adverse event (Table 3). Early adverse reactions included allergic reactions in seven patients (3.65%) and infusion-related headaches in 13 (6.77%). Late adverse events included infections requiring IV antibiotics in 18 patients (9.38%), oral ulcers in 12 (6.25%), gastritis in 15 (7.81%), elevated liver function tests in 6 (3.13%), and lipid abnormalities in 19 patients (9.90%).

**Table 3:** Adverse Events Observed During the Six-Month Follow-Up (n = 192). Values presented as n (%).

Category	Adverse Event	Patients n (%)
Early Adverse Reactions	Allergic Reaction (within 24 hrs)	7 (3.65)
	Infusion-related Headache	13 (6.77)
Late Adverse Events	Infections (requiring IV antibiotics)	18 (9.38)
	Oral Ulcers	12 (6.25)
	Gastritis	15 (7.81)
	Elevated LFTs (ALT/AST >2× ULN)	6 (3.13)
	Lipid Abnormalities (TG or LDL)	19 (9.90)
Overall	Any Adverse Event	52 (27.08)

Baseline labs revealed hemoglobin levels below normal in 81 patients (42.19%), abnormal total leukocyte count in 17 (8.85%), and low platelet count in 6 (3.13%), as shown in Table 4. Elevated alanine transaminase (ALT) was seen in 21 patients (10.94%), high serum creatinine in 9 (4.69%), raised low-density lipoprotein (LDL) cholesterol in 52 (27.08%), and elevated triglycerides in 46 (23.96%).

**Table 4:** Baseline Laboratory Investigations of Study Participants (n = 192). Values are shown as Mean ± SD or n (%) abnormalities. ALT: alanine transaminase, LDL: low-density lipoprotein

Parameter	Mean ± SD	Normal Range	Abnormal n (%)
Hemoglobin (g/dL)	11.9 ± 1.4	12–16 (F), 13–17 (M)	81 (42.19)
Total Leukocyte Count (/cmm)	6,420 ± 1,330	4,000–11,000	17 (8.85)
Platelet Count (/cmm)	296,000 ± 70,500	150,000–450,000	6 (3.13)
ALT (U/L)	39 ± 18	<40	21 (10.94)
Serum Creatinine (mg/dL)	0.88 ± 0.17	0.6–1.2	9 (4.69)
LDL Cholesterol (mg/dL)	138 ± 27	<130	52 (27.08)
Triglycerides (mg/dL)	162 ± 45	<150	46 (23.96)

Laboratory abnormalities increased over time. At baseline, 21 patients (10.94%) had elevated liver function tests (LFTs), 52 (27.08%) had lipid abnormalities, and 92 (47.92%) had complete blood count (CBC) abnormalities (Table 5). At week 12, these increased to 25 (13.02%) for LFTs, 68 (35.42%) for lipids, and 78 (40.63%) for CBC. By week 24, LFT abnormalities were seen in 29 patients (15.10%), lipid issues in 74 (38.54%), and CBC abnormalities decreased to 65 patients (33.85%).

**Table 5:** Longitudinal Laboratory Abnormalities During the Study Period (n = 192). Values presented as number of patients (percentage). LFT: liver function test; CBC: complete blood count.

Time Point	LFT Abnormalities n (%)	Lipid Abnormalities n (%)	CBC Abnormalities n (%)
Baseline	21 (10.9)	52 (27.1)	92 (47.9)
Week 12	25 (13.0)	68 (35.4)	78 (40.6)
Week 24	29 (15.1)	74 (38.5)	65 (33.9)

Paired t-test comparisons showed significant reductions in disease activity (Table 6). From baseline to week 12, DAS28 decreased by a mean of 2.15 points (95% CI: 2.06-2.24; p < 0.001). From baseline to week 24, the reduction was 3.48 points (95% CI: 3.35-3.61; p < 0.001). Additionally, from week 12 to week 24, the mean DAS28 further reduced by 1.33 points (95% CI: 1.21-1.45; p < 0.001), confirming consistent and statistically significant improvement in all 192 patients.

**Table 6:** Paired t-test Analysis of DAS28 Scores at Baseline, Week 12, and Week 24 (n = 192). Results are expressed as mean difference with 95% confidence interval (CI). p < 0.05 is considered statistically significant.

Comparison	Baseline Mean ± SD	Follow-up Mean ± SD	Mean Difference	95% CI	p-value
Baseline vs. Week 12	5.91 ± 0.42	3.76 ± 0.59	2.15	2.06 – 2.24	<0.001
Baseline vs. Week 24	5.91 ± 0.42	2.43 ± 0.71	3.48	3.35 – 3.61	<0.001
Week 12 vs. Week 24	3.76 ± 0.59	2.43 ± 0.71	1.33	1.21 – 1.45	<0.001

## Discussion

The current trial looked at the safety and effectiveness of tocilizumab in 192 individuals with RA who did not respond to traditional synthetic DMARDs (csDMARDs). The results showed a substantial improvement in clinical symptoms. The average DAS28 score dropped from 5.91 ± 0.42 at the start to 3.76 ± 0.59 at 12 weeks and then to 2.43 ± 0.71 at 24 weeks (p < 0.001), which shows that the treatment worked well. By six months, 112 patients (58.33%) had gone into remission, and 58 more (30.21%) had had a partial response. These results are in line with what other trials have found: 40% of patients who had tocilizumab treatment for 24 weeks went into remission [15].

Our results are quite similar to those of prior research that found a mean DAS28 decrease in individuals who were given tocilizumab [16]. Also, prior research showed that treating RA patients with tocilizumab resulted in high remission rates at six months when measured with the DAS28 score, although these rates were lower than the 58.33% remission rate we found in our study [17]. This discrepancy might be because our trial included a younger group of patients (56.25% were 45 years old or younger), a larger percentage of female patients (77.08%), and everyone followed a normal dose schedule.

The safety review showed that 52 patients (27.08%) had bad outcomes, including 18 (9.38%) who had infections that needed intravenous antibiotics, 19 (9.90%) who had cholesterol problems, and 13 (6.77%) who had headaches connected to the infusion. These rates are in line with other observational studies that found a 23% rate of infections during a comparable time frame [18].

Baseline test problems, such as anemia (42.19%) and leukocytosis (8.85%), were comparable to what is seen in registry-based studies of the general RA population [19]. Long-term monitoring indicated that CBC values improved over time, which might be due to the anti-inflammatory effects of tocilizumab. However, in week 24, 15.10% of patients had elevated liver enzymes, which meant that continuing hepatic monitoring was necessary, as the previous research [20] stressed.

Overall, our data demonstrates that tocilizumab works quite well for those with RA who don't respond well to traditional synthetic DMARDs. The study's results, which showed a big drop in DAS28 values and high remission rates, show that this treatment might be very useful in everyday clinical practice. Also, its good tolerability profile makes it more likely to be used in real life. The results also show how important it is to keep an eye out for any side effects, including infections, cholesterol problems, and liver problems, which may happen during biologic treatment and need quick medical attention.

Strengths and limitations

A major strength of this study is its prospective design and relatively large sample size (n = 192), which enhances the reliability and generalizability of the findings to comparable clinical settings. The use of standardized inclusion criteria, regular follow-up intervals, and objective disease activity assessment with DAS28 further strengthens the validity of the results. Importantly, there is limited real-world evidence on tocilizumab use in South Asian populations, making this study a valuable contribution to the literature.

The study also has limitations. Being single-center and based on convenience sampling, there is potential for selection bias. The absence of a control or comparator group limits the ability to attribute observed improvements exclusively to tocilizumab. Furthermore, the follow-up period of six months, while sufficient to capture short-term efficacy and safety, may not reflect long-term outcomes such as sustained remission, radiographic progression, or delayed adverse effects. Finally, although an observational cohort design provides valuable real-world insights, it lacks the inferential strength of randomized controlled or case-control studies, as noted by reviewers.

## Conclusions

This prospective research shows that tocilizumab is very good at lowering disease activity and putting patients with RA who don't respond to standard DMARDs into remission. A total of 58.33% of patients achieved remission, and there was a statistically significant improvement in DAS28 values. Even though 27.08% of patients had bad events, most of them were controllable, which shows that the safety profile is good. These results support the use of tocilizumab as a strong treatment choice in everyday clinical practice for patients who don't respond well to csDMARDs. They also stress the need for ongoing monitoring for lab and infectious problems.
